# Second-harmonic electron paramagnetic resonance spectroscopy and imaging reveal metallic lithium depositions in Li-ion batteries

**DOI:** 10.5194/mr-5-87-2024

**Published:** 2024-06-25

**Authors:** Charles-E. Dutoit, Hania Ahouari, Quentin Denoyelle, Simon Pondaven, Hervé Vezin

**Affiliations:** 1 Université Lille Nord de France, CNRS, UMR8516, LASIRE, 59655 Villeneuve d'Ascq, France; 2 Centre de Résonance Magnétique Electronique pour les Matériaux et l'Energie, Université Lille Nord de France, 59655 Villeneuve d'Ascq, France; 3 Université de Lille, FR2638, IMEC, Institut Michel-Eugène Chevreul, 59655 Villeneuve d'Ascq, France; 4 SAFT, Corporate Research, 111 Boulevard Alfred Dancy, 33074 Bordeaux, France; 5 TotalEnergies OneTech R&D, Centre de Recherche de Solaize (CRES), Chemin du Canal, BP 22, 69360 Solaize, France

## Abstract

We have investigated metallic lithium particle nucleation following lithiation and delithiation steps of the graphite electrode using X-band electron paramagnetic resonance (EPR). Metallic lithium aggregates like dendrites and/or filaments which are formed during electrochemical cycling on the graphite anode are complex structures which may lead to internal short-circuit and safety issues. Understanding and following, in real conditions, this nucleation process is necessary to improve the development of Li-ion batteries. The complexity to detect metallic lithium structures inside Li-ion batteries depends on the number of EPR lines and their linewidth. The presence of lithiated graphite phases affects the detection of micrometric Li-metal elements. Herein, we report a new approach using cw-EPR (continuous-wave EPR) spectroscopy and imaging, combining the first- and second-harmonic detection schemes to provide evidence for the metallic lithium aggregate nucleation in these negative electrodes. Although the first harmonic gives all the EPR signals present in the sample, it is found that the second-harmonic EPR signal is mainly sensitive to metallic lithium depositions.

## 1 Introduction

The family of rechargeable Li-ion batteries (LIBs) is known to be used in a wide variety of applications, from portable electronics to electric vehicles, due to their high specific capacities, good lifespan and their decreasing cost [Bibr bib1.bibx22]. More particularly, with their high capacities of 372 mAh g ${}^{-1}$ , graphite electrode materials are the most widely used anodes in such batteries. Due to the low-voltage open-circuit potential of graphite, metallic lithium is susceptible to deposit onto the graphite particles at the negative electrode during charging of the cell, i.e. during the graphite lithiation. This parasitic reaction is more likely to happen at low temperature, high charge rates and a high state of charge. In normal operating conditions, the lithium plating level is low but participates in cell capacity loss due to lithium consumption in the metallic aggregates and in the formation of the additional solid electrolyte interphase (SEI). For more severe charge conditions, lithium can even form dendrites, which can lead to safety issues in the case of internal short circuits. The non-uniform metallic lithium plating on the graphite anode is the main limitation for a faster charge protocol, essential for the development of transport electrification [Bibr bib1.bibx13]. Following this degradation process in real time and with operating conditions is challenging yet necessary to keep improving the Li-ion battery performance [Bibr bib1.bibx10].

This type of information requires the use of non-destructive methods which leave the sample intact during and after measurements, without destroying sub-micrometric metallic aggregates newly formed. Magnetic resonance techniques appear to be suitable for ex situ, in situ and operando measurements of Li-metal structures due to their low-frequency fields using radio and microwave frequency for nuclear magnetic resonance (NMR) and electron paramagnetic resonance (EPR), respectively, which penetrate samples with negligible energies.

NMR spectroscopy and imaging are well-established techniques to investigate redox processes in Li-based electrochemical batteries but also to detect metallic particles which can grow during the charge and discharge processes [Bibr bib1.bibx2]. EPR, which is the electronic equivalent of NMR, is the most convenient method to probe in-depth Li-metal depositions like the bulk, dendrites or metallic filaments through its high sensitivity to conduction electrons [Bibr bib1.bibx19], when compared to NMR spectroscopy. However, in the case of graphite (de)lithiation, the resolution of the Li-metal EPR spectrum is limited by the presence of broad lithiated graphite signals at a resonance field near the one of Li metal. As a consequence, the Li-metal signal is overlapping with the lithiated graphite spectrum and is sometimes not recognizable.

In this work, we report the direct observation of metallic lithium depositions with micrometric sizes on the negative graphite electrode using, for the first time, the second-harmonic detection mode. We correlate the first- and second-harmonic detection schemes of EPR spectroscopy and imaging to obtain information about the nucleation and the spatial distribution of metallic lithium structures in LiFePO ${}_{4}$ / graphite batteries.

## 2 Experimental details

### 2.1 Electrochemical cell

In this study, a LiFePO ${}_{4}$ (LFP) / graphite cell was considered using a LiPF ${}_{6}$ lithium salt dissolved in carbonate solvents. After the usual formation cycles, cycling of the cell was carried out at 20 °C with a regime of C/2 (theoretically one Li per LiFePO ${}_{4}$ unit in 2 h) performed on 90 % of the state of charge (SOC) of the cell. The aged cell was cycled several thousand times until 30 % of capacity loss. The cell was then discharged and dismantled in an argon-filled glove box. The graphite electrode was washed three times using dimethyl carbonate (DMC) and dried. The anode was sampled by cutting out rectangles taken from the center of the anode and then placed in a sealed tube before EPR analysis.

### 2.2 Electron paramagnetic resonance

Continuous-wave (cw) electron paramagnetic resonance measurements were carried out at room temperature using a conventional X-band Bruker E500 spectrometer operating at around 9.6 GHz. The microwave power into the cavity was set to 0.2 mW in order to avoid saturation of the EPR signal. The 100 kHz modulation depth of the magnetic field was chosen as 0.2 mT or less to prevent distortion of the apparent EPR spectrum due to over-modulation. Conversion time and time constant were set to 40.94 and 20.48 ms, respectively. Simulation of cw-EPR spectra was done using the EasySpin package for MATLAB [Bibr bib1.bibx21]. The first-harmonic spectrum was fitted using the sum of the two phase-shifted Lorentzian functions defined in Eq. ([Disp-formula Ch1.E1]). The asymmetric ratio A/B of the EPR line was obtained by considering the first-derivative EPR spectrum for lithiated graphite signal and the second derivative EPR spectrum for the metallic lithium aggregates.

Spatial–spatial and spectral–spatial images were collected using a field of view of 20 mm and a gradient strength of 175 G cm ${}^{-1}$ (where $1\;G=10^{-4}\;T$ ) with a size of 512×512 pixels, resulting in a pixel size of 39.1 µ m. The high-resolution spatial–spatial images were recorded at room temperature using a deconvolution of the acquired projections under a magnetic field gradient from a signal recorded without gradient. Finally, EPR images were filtered with a back-projection; 330 projections were recorded in the spectral–spatial images with a spectral resolution of 1024 points and a pixel size similar to the one for spatial–spatial images. A filtered back-projection of the acquired projections was performed to get high-resolution images for signals with a peak-to-peak linewidth lower than 10 G.

## 3 Results and discussion

The reversible lithiation and delithiation of the graphite anode during a C/2 charge rate is analyzed at room temperature to provide evidence or not for metallic lithium traces in our LiFePO ${}_{4}$ / graphite battery. Galvanostatic discharge curves are shown in Fig. [Fig Ch1.F1]a for the first and the last cycle before opening. As expected for a LFP / graphite cell, the potential is nearly constant during all of the discharge, due to the flat open-circuit potentials of LFP and graphite. At the beginning of life, the graphite plateaus are well visible but almost disappear at the end of life. Indeed, for these cells the main aging phenomenon is the solid electrolyte interphase (SEI) buildup, coming with loss of active lithium and graphite polarization increase. Figure [Fig Ch1.F1]b shows a representative X-band cw-EPR signal of the graphite anode recorded before electrochemical cycling (black line). The spectrum exhibits a single and broad Dysonian EPR line with a g factor of about 2.01 (resonance field of 341.4 mT at 9.61 GHz) and a peak-to-peak linewidth $\Delta B_{\mathrm{pp}}∼3$ mT. It is well known that the g factor of a radical is characterized by a specific environment (similar to the chemical shift in NMR spectroscopy). Typically, a g factor of 2.01 may be attributed to an oxygen-centered radical. Such an EPR signal is classically found in graphite electrode materials [Bibr bib1.bibx27]. As expected, initially the metallic lithium EPR signal is featureless, consistent with pure graphite materials without Li-metal impurities.

**Figure 1 Ch1.F1:**
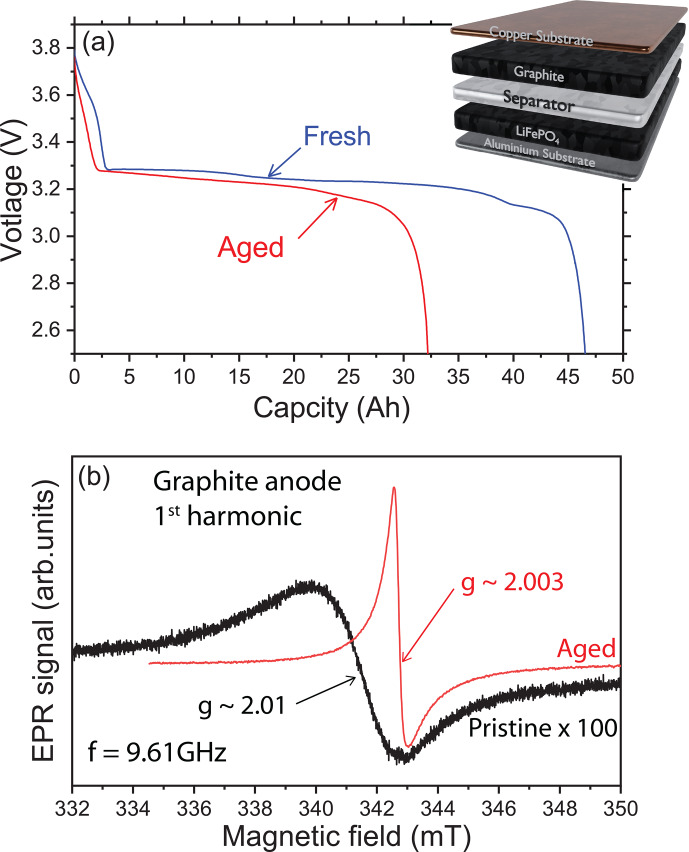
X-band cw-EPR spectroscopy of the graphite anode. **(a)** Galvanostatic cycling profile of the LiFePO ${}_{4}$ / graphite cell with a schematic representation of the electrode material stacking. The blue curve (“Fresh”) is the first discharge and the red curve (“Aged”) is the last discharge of the cell. **(b)** EPR spectra of the pristine (black) and aged (red) electrodes using the first-harmonic detection scheme. Note that the black curve has been amplified ( ×100 ) to compare the signal intensities.

An example of the ex situ EPR spectrum of the graphite anode after electrochemical cycling is given in Fig. [Fig Ch1.F1]b (red line). The shape of the EPR signal displays a different general pattern compared to the pristine spectrum with a line centered at a value g∼2.0036 , smaller than the one observed in the pristine state. Furthermore, the EPR line appears narrower than the pure graphite signal. Also, this spectrum is characterized by a Dysonian line more intense than in the pristine sample considering the same mass of matter. It can be seen that this signal has a peak-to-peak linewidth of about 0.3 mT and an asymmetric ratio A/B ∼2 , as expected for the lithiated graphite [Bibr bib1.bibx26]. Indeed, the cycled cell was discharged before opening, but it corresponds to a state where the graphite is still partially lithiated.

In Fig. [Fig Ch1.F2], we represent a simulation of the lithiated graphite signal considering two different cases. In the first one, a first derivative of a single Dysonian function is used to simulate the EPR spectrum. As we can see, the main features of the experimental spectrum are not correctly reproduced, suggesting at least another additional contribution hidden under the lithiated graphite spectrum. The ability to resolve this second contribution depends on the number of functions used in the simulation. In the second case, the signal was simulated using a sum of two contributions: (i) a relative broad Dysonian function with an asymmetric ratio A/B∼1.6 and a linewidth ΔB∼1 mT for the lithiated graphite species Li ${}_{x}$ C ${}_{6}$ ( 0<x≤1 ); (ii) a narrow Dysonian line with A/B ∼1.8 and ΔB∼0.2 mT. It is worth noting that this second EPR line is possibly over-modulated due to the modulation amplitude of 0.2 mT used in this experiment. The modulation amplitude value of 0.2 mT was chosen due to the apparent peak-to-peak linewidth of the spectrum observed before analysis (around 1 mT). Consequently, the real linewidth of this second signal is necessarily smaller than 0.2 mT. This additional EPR line is assigned to traces of metallic lithium aggregates with a size slightly larger than the skin depth (non-dendritic); here $\delta_{\mathrm{mw}}$ ∼1.1 µ m at 9.6 GHz. Indeed, as discussed in a previous EPR investigation of symmetric Li-metal/Li-metal cells [Bibr bib1.bibx5], the EPR lineshape is influenced by the metal thickness compared to $\delta_{\mathrm{mw}}$ due to the excitation of spins located exclusively inside the skin depth. When the metal thickness is greater than $\delta_{\mathrm{mw}}$ , a Dysonian EPR lineshape is observed with an asymmetric ratio A/B≫1 [Bibr bib1.bibx6]. In contrast, if the metal thickness is smaller than $\delta_{\mathrm{mw}}$ , a pure Lorentzian line is obtained with an asymmetric ratio A/B=1 . It is important to note that the EPR spectrum of the graphite anode recorded after the first half lithiation (electrode potential ∼86 mV) does not show a distinguishable Li-metal contribution (see Supplement Fig. S1).

**Figure 2 Ch1.F2:**
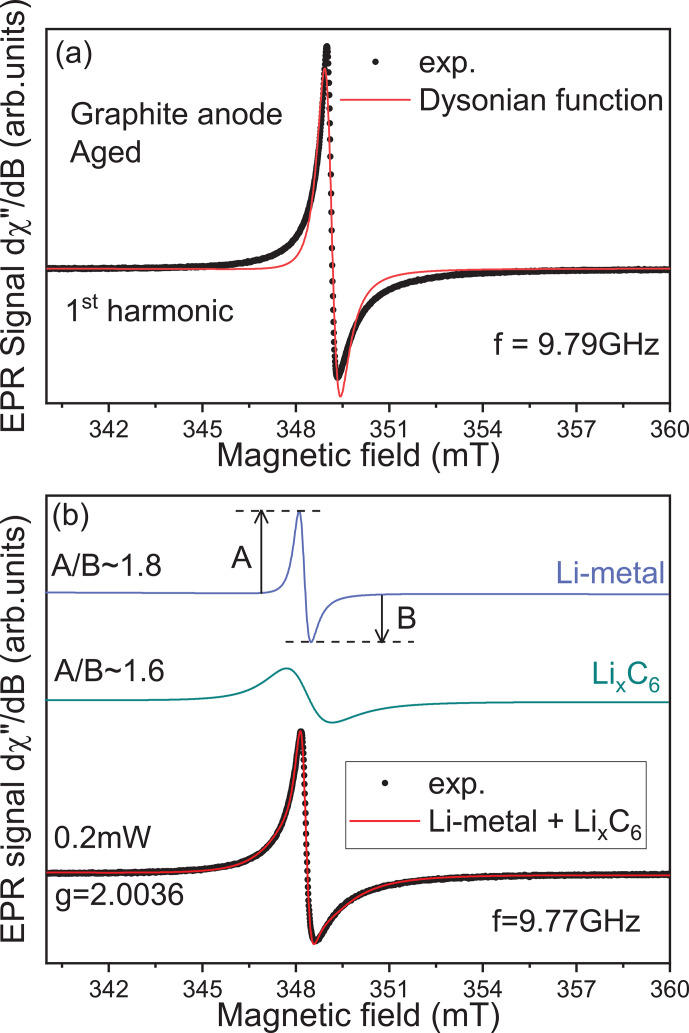
Identification of the second EPR contribution hidden under the lithiated graphite signal. **(a)** Simulation of the lithiated graphite anode aged signal recorded at room temperature exclusively using one Dysonian function. **(b)** Same simulation using two Dysonian contributions. The green line represents the lithiated graphite spectrum (Li ${}_{x}$ C ${}_{6}$ ), and the blue line is indicative of Li metal.

No rigorous theoretical models for porous metallic lithium micro-particles are available, and we chose to estimate the smallest metallic lithium particle size from the empirical equation [Bibr bib1.bibx11] defined by

1 $$\frac{dP}{dB}=\alpha \frac{1-\chi^{2}}{(1+\chi^{2}{)}^{2}}-\beta \frac{2\chi }{(1+\chi^{2}{)}^{2}},$$

with $\chi =\gamma (B-B_{\mathrm{res}})T_{2}$ , γ representing the electron gyromagnetic ratio, and α and β being the dispersion and absorption parameters, respectively. From this expression and the parameters α and β characterizing the lineshape (and thus the asymmetric ratio A/B ), we estimate a micrometric size of about 1.6 µ m.

**Figure 3 Ch1.F3:**
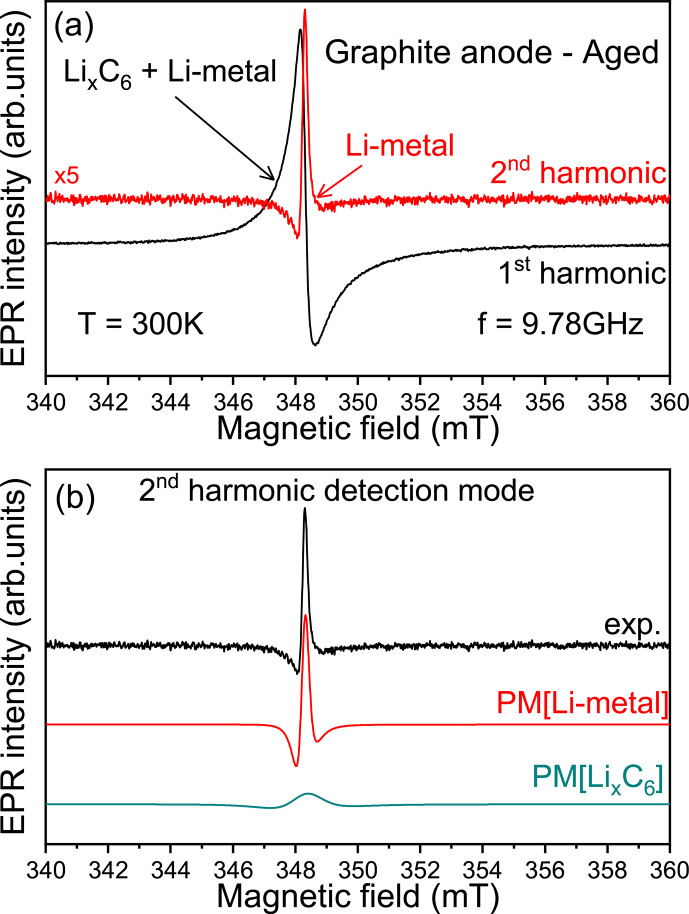
Second-harmonic X-band EPR detection scheme. **(a)** EPR spectra of lithiated graphite anode aged signal recorded at room temperature using the first- (black) and second-harmonic (red) detection modes. **(b)** Pseudo-modulated (PM) EPR spectra recorded from the simulated signals Li metal (“PM[Li-metal]”) and Li ${}_{x}$ C ${}_{6}$ (“PM[Li ${}_{x}$ C ${}_{6}$ ]”) defined in Fig. [Fig Ch1.F2].

**Figure 4 Ch1.F4:**
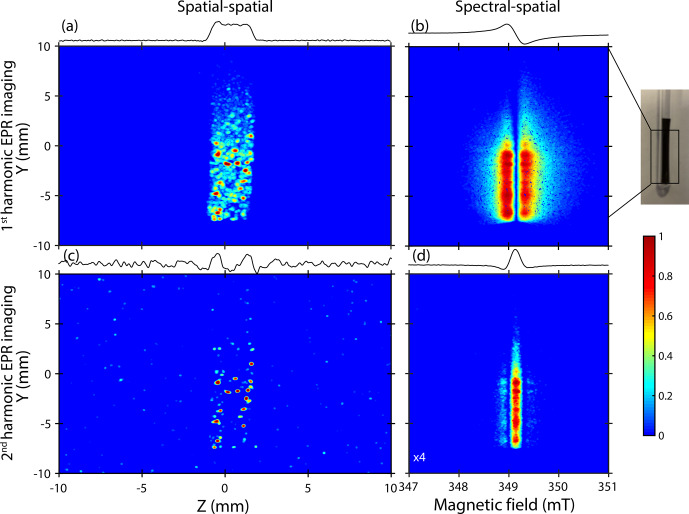
X-band EPR detection and location of Li-metal depositions. **(a–b)** Spatial–spatial and spectral–spatial images of the lithiated graphite anode recorded using the standard detection mode (first harmonic). **(c–d)** Second-harmonic spatial–spatial and spectral–spatial images. For clarity, the spin contribution of the spectral–spatial image is indicated by the absolute value of the EPR spectrum where the red color represents the positive and negative lobes. The color code is indicated by the color bar and illustrates the apparent amplitude of the signals.

In summary, the cw-EPR signal of lithiated graphite samples, recorded at the X-band, contains a mixture of two overlapping contributions, i.e., lithiated graphite complexes and traces of micrometric metallic lithium aggregates. In the absence of rigorous simulations, such metallic signals could be indistinguishable. Furthermore, the difficulty in distinguishing such traces of non-dendritic Li metal at low potential due to the weaker Li-metal line that is masked by the intense Li ${}_{x}$ C ${}_{6}$ peak has been already reported in the literature [Bibr bib1.bibx27]. To clearly observe such metallic structures in EPR measurements, we have to improve the spectral selectivity. We suggest here a new approach by playing with the harmonic detection schemes of the EPR spectrum [Bibr bib1.bibx20]. In a conventional X-band EPR spectrometer, there is an option which allows for the detection of the second harmonic of the modulated EPR spectrum, i.e., the second derivative of the absorption signal (simultaneously with the first-harmonic mode). The first-harmonic mode (first derivative) is routinely used in standard EPR and gives EPR signals of all magnetic species present in the sample, here the lithiated graphite and the Li-metal signals. The second harmonic, which is mainly used for resolution enhancement of unresolved hyperfine structures, gives a better spectral resolution for overlapping EPR signals. Indeed, with the n th harmonic being sensitive to the slope of the EPR signal, i.e. sensitive to the spin–spin relaxation time $T_{2}$ [Bibr bib1.bibx18], a broader spectrum tends to be less prominent than a sharper peak with higher-order harmonics without spectral distortion caused by slight over-modulation [Bibr bib1.bibx29]. Figure [Fig Ch1.F3] shows an example of X-band spectra obtained after the galvanostatic cycling and recorded using the first- (black) and second-harmonic (red) detection modes. As discussed previously, the first harmonic mainly reveals the presence of lithiated graphite (no Li-metal signal directly distinguishable). As we can see, the second-harmonic spectrum exclusively contains one contribution centered, in the limit of the X-band spectrometer resolution, at a similar measured resonance field than the lithiated graphite. Furthermore, this signal displays a very sharp Dysonian EPR line consistent with the Li-metal signal. This interpretation is reinforced by the results presented in Fig. [Fig Ch1.F3]b. As we can see, the pseudo-modulation (PM) of the simulated EPR signals, corresponding to the Li-metal and Li ${}_{x}$ C ${}_{6}$ defined in Fig. [Fig Ch1.F2], displays two different behaviors. Although the signal of PM[Li ${}_{x}$ C ${}_{6}$ ] shows a large and flattened line, the signal of PM[Li-metal] is intense and in good agreement with the second-harmonic line recorded experimentally. We tested the second-harmonic spectrum assignment by using a symmetric cell with metallic lithium disks as both electrodes (see Fig. S2). Initially, the first-harmonic EPR signal consists of a broad Dysonian lineshape characteristic of the bulk lithium signal. After the short circuit, the EPR spectrum exhibits two contributions: (i) a large Dysonian line corresponding to the Li-metal electrode (bulk) and (ii) a very sharp spectrum characteristic of sub-micrometric metallic Li particles. Finally, the second harmonic contains only the Li-metal sub-micrometric information and shows a similar shape and g factor to the one obtained in Fig. [Fig Ch1.F3]a, confirming our hypothesis. These results show that in the case of the graphite lithiation the metallic aggregates formed during electrochemical cycling are better resolved in this detection mode.

Now, let us discuss the spatial distribution of these metallic particles. Figure [Fig Ch1.F4] focuses on the ex situ EPR images recorded on cycled samples. A gradient of 175 G cm ${}^{-1}$ was used for spatially encoding both complexes (lithiated graphite and Li metal) with a high resolution due to their respective linewidths ( ≤10 G), especially for metallic lithium element signals which display a peak-to-peak linewidth of about 1 G [Bibr bib1.bibx17]. In these examples, we used the spatial–spatial detection mode to get information about the location of aggregates in two perpendicular spatial directions Y and Z . Furthermore, we correlated the spatial–spatial images with spectral–spatial images to obtain spectroscopic information (lineshape, resonance field, asymmetric ratio A/B , etc.). These spectroscopic parameters are crucial to clearly validate the nature and the origin of each signal visible in EPR images [Bibr bib1.bibx5].

Figure [Fig Ch1.F4]a–b show the EPR images recorded using the spatial–spatial and the spectral–spatial detection schemes, respectively. The sample appears in the center from the intense EPR signals, and its apparent shape is similar to the real shape with dimensions of about 25mm×2.5mm . The spatial–spatial image confirms the non-homogeneous distribution of lithiated graphite species with spots mainly located on the bottom part of the sample. Some additional very intense signals (red) displaying a non-uniform distribution are clearly visible. This result suggests that some aggregates are more sensitive to the microwave field $b_{\mathrm{mw}}$ . The corresponding spectral–spatial image confirms the presence of lithiated graphite species characterized by a relative broad Dysonian EPR shape centered at a g value of ∼2.0036 and displaying a peak-to-peak linewidth of about 0.3 mT (Fig. [Fig Ch1.F4]b).

In order to distinguish and locate metallic lithium structures in the sample, we introduced here the correlation between the first- and second-harmonic EPR images. This approach is new to the best of our knowledge. As shown before, in our electrochemical system, the second-harmonic EPR spectrum is exclusively sensitive to the metallic lithium aggregates. Figure [Fig Ch1.F4]c shows the second-harmonic spatial–spatial image of the same sample. Intense spots observed here seem to be correlated with those initially found in the standard detection mode. This result is the indication of Li-metal nucleation at the graphite anode surface and confirms that these aggregates are mainly located near the lithiated graphite regions. It is worth noting that the pixel size used for recording the EPR images is around 39.1 µ m, which does not allow us to clearly visualize the particles with a dimension close to 1.6 µ m estimated by EPR spectroscopy.

## 4 Conclusions

To conclude, EPR spectroscopy is a nondestructive, rapid and sensitive method to detect micrometric and/or sub-micrometric Li-metal elements. The aim of this investigation was to monitor the metallic lithium aggregate nucleation on the graphite anode following lithiation and delithiation steps using multi-mode EPR spectroscopy and imaging. It was shown that the second-harmonic detection scheme is sensitive to the Li-metal structures with a size slightly larger than the skin depth. This effect allows to distinguish the spectroscopic signature of the metallic element when it is overlapping with the lithiated graphite signal. We provide the correlation between the first- and second-harmonic detection modes of EPR spectroscopy and EPR imaging to follow the Li-metal deposition. To date, and to the best of our knowledge, the second-harmonic detection mode was never used to clearly distinguish Li-metal plating/stripping in graphite electrodes. This result offers an alternative approach for Li-based batteries, paving the way for the detection and location of Li-metal aggregates.

## Supplement

10.5194/mr-5-87-2024-supplement The supplement related to this article is available online at: https://doi.org/10.5194/mr-5-87-2024-supplement.

## Supplement

10.5194/mr-5-87-2024-supplement
10.5194/mr-5-87-2024-supplement The supplement related to this article is available online at: https://doi.org/10.5194/mr-5-87-2024-supplement.

## Data Availability

The dataset that supports the findings of this investigation is available here: 10.5281/zenodo.10623150 [Bibr bib1.bibx4].

## Code availability

MATLAB is a commercial software from MathWorks, and the EasySpin package for EPR simulation is available at https://www.easyspin.org/download.html ([Bibr bib1.bibx21]).

## Appendix

The supplement related to this article is available online at: https://doi.org/10.5194/mr-5-87-2024-supplement.
