# Supplementary material for: Second-harmonic electron paramagnetic resonance spectroscopy and imaging reveal metallic lithium depositions in Li-ion batteries
Source: Magn Reson (Gott). 2024 Jun 25;5(1):87–93. doi: 10.5194/mr-5-87-2024 (PMC12082685; doi:10.5194/mr-5-87-2024)

Supplement of Magn. Reson., 5, 87–93, 2024
https://doi.org/10.5194/mr-5-87-2024-supplement
© Author(s) 2024. CC BY 4.0 License.

Supplement of

Second-harmonic electron paramagnetic resonance spectroscopy and imaging reveal metallic lithium depositions in Li-ion batteries

Charles-E. Dutoit et al.

Correspondence to: Charles-E. Dutoit (charles.dutoit@univ-lille.fr)

The copyright of individual parts of the supplement might differ from the article licence.

Supplementary Figure S1: 1st harmonic X-band EPR spectrum of graphite anode recorded after the first half lithiation (electrode potential of 86mV). The red line represents the simulation using a single dysonian function and shows that the experimental spectrum is correctly reproduced suggesting only one contribution.

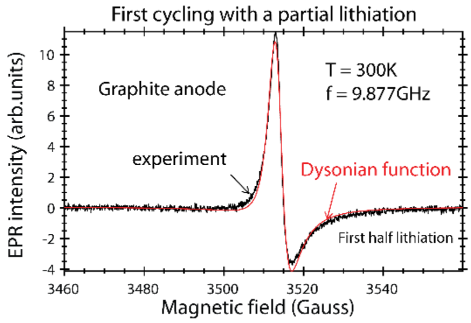

Supplementary Figure S2: a Voltage and current of the symmetric Li-metal electrochemical cell as a function of the cycling time. **b** Set of X-band EPR spectra of the cell recorded before cycling (i) and after the short circuit (ii). Both EPR signals (ii) were obtained using the 1st and the 2nd harmonic detection scheme respectively.

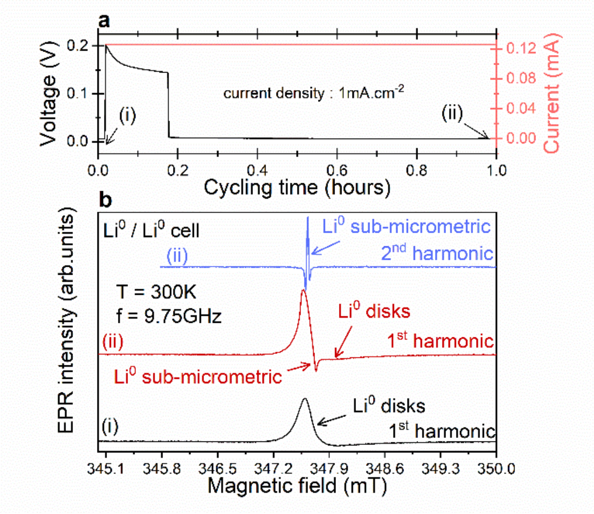

Supplement: The supplement related to this article is available online at: https://doi.org/10.5194/mr-5-87-2024-supplement. [file mr-5-87-2024-supplement.pdf]
